# Thrombocytopenia and endocarditis in a patient with Whipple’s disease: case report

**DOI:** 10.1186/s12879-020-4799-0

**Published:** 2020-01-22

**Authors:** Maxim Olivier, Carmelo Licitra, Zachary Field, Li Ge, Dustin Hill, Mario Madruga, S. J. Carlan

**Affiliations:** 1 Department of Medicine, Orlando Regional Healthcare, Orlando, Florida USA; 2 Division of Infectious Diseases, Orlando Regional Healthcare, Orlando, Florida USA; 3 Department of Pathology, Orlando Regional Healthcare, Orlando, Florida USA; 4 Division of Cardiology, Orlando Regional Healthcare, Orlando, Florida USA; 5 Division of Academic Affairs and Research, Orlando Regional Healthcare, 1401 Lucerne Terrace, 2nd floor, Orlando, Fl 32806 USA

**Keywords:** Thrombocytopenia, Whipple’s disease, Endocarditis, Embolic stroke, Malabsorption

## Abstract

**Background:**

Whipple’s disease (WD) is a rare multisystem infectious disorder that is caused by the actinomycete *Tropheryma whipplei.* It presents with joint pain followed by abdominal pain, diarrhea, malabsorption and finally failure to thrive. Diagnosis requires tissue sampling and histology with periodic acid-Schiff [PAS] staining. Thrombocytopenia associated with endocarditis associated with WD has been reported twice.

**Case presentation:**

A 56 year old Caucasian male presented with years of steroid treated joint pain and recent onset diarrhea, weight loss and abdominal pain. Ultimately he was found to have a platelet count of 4000 with concomitant endocarditis and embolic stroke. Small bowel biopsy confirmed the diagnosis of WD approximately 1 year after his first visit. His platelets improved with antibiotic treatment but he eventually expired 16 months after his initial consult and 5 months after his definitive diagnosis.

**Conclusion:**

WD can remain undiagnosed and untreated until late in the course of the illness. A high index of suspicion is recognized as necessary for early diagnosis to begin treatment. Critical thrombocytopenia associated with endocarditis is a rare and potentially poor prognostic sign in late stage Whipple’s disease.

## Background

Whipple’s disease (WD) is a rare multisystem infectious disorder characterized by joint pain, diarrhea, abdominal pain, failure to thrive and progressive wasting. It is caused by the actinomycete *Tropheryma whipplei* which is ubiquitous in the environment. The diagnosis typically requires polymerase chain reaction (PCR) testing, followed by immunohistochemistry and confirmation with histology with periodic acid-Schiff [PAS] staining of infected tissue. The treatment includes an initial phase of an intravenous antibiotic that is active against *T. whipplei* and is known to penetrate the blood-brain barrier, followed by 12 months of oral maintenance therapy. Involvement of the cardiopulmonary, neuropsychiatric and even hematopoietic systems in WD have been described. Laboratory changes associated with chronic inflammation and malabsorption have been reported, as well [1]. Anemia, neutrophilia and thrombocytosis are common findings. Extremely rare, however, is thrombocytopenia. There have been two previous reports [2, 3] of moderate thrombocytopenia associated with WD endocarditis. We present a case of WD associated with blood culture negative endocarditis and a platelet count of 4000.

## Case presentation

**Fig. 1:**
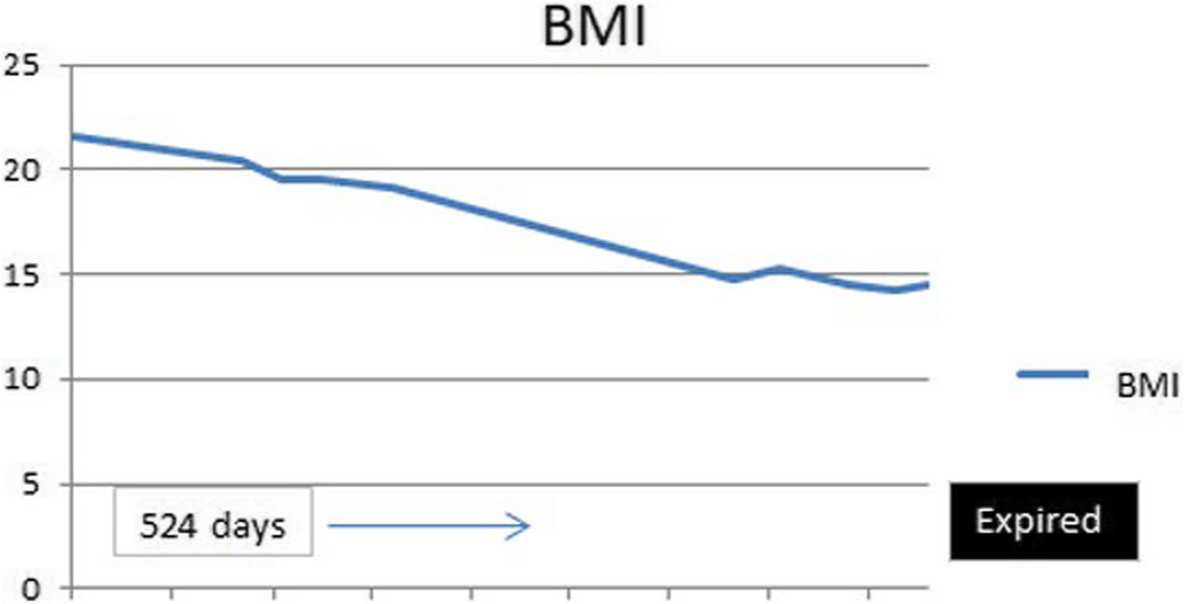
Weight loss over time documented for 524 days

**Fig. 2:**
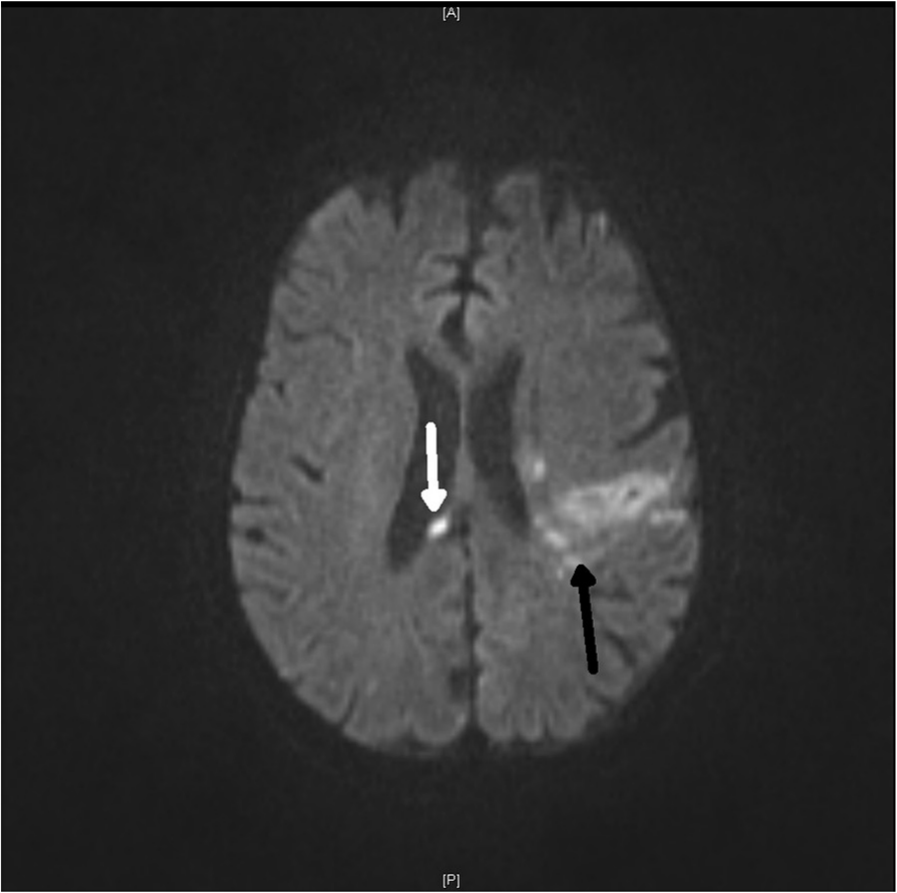
MRI head shows a left temporal infarct (black arrow) and a small R sided periventricular infarct (white arrow). Findings consistent with embolic stroke

**Fig. 3:**
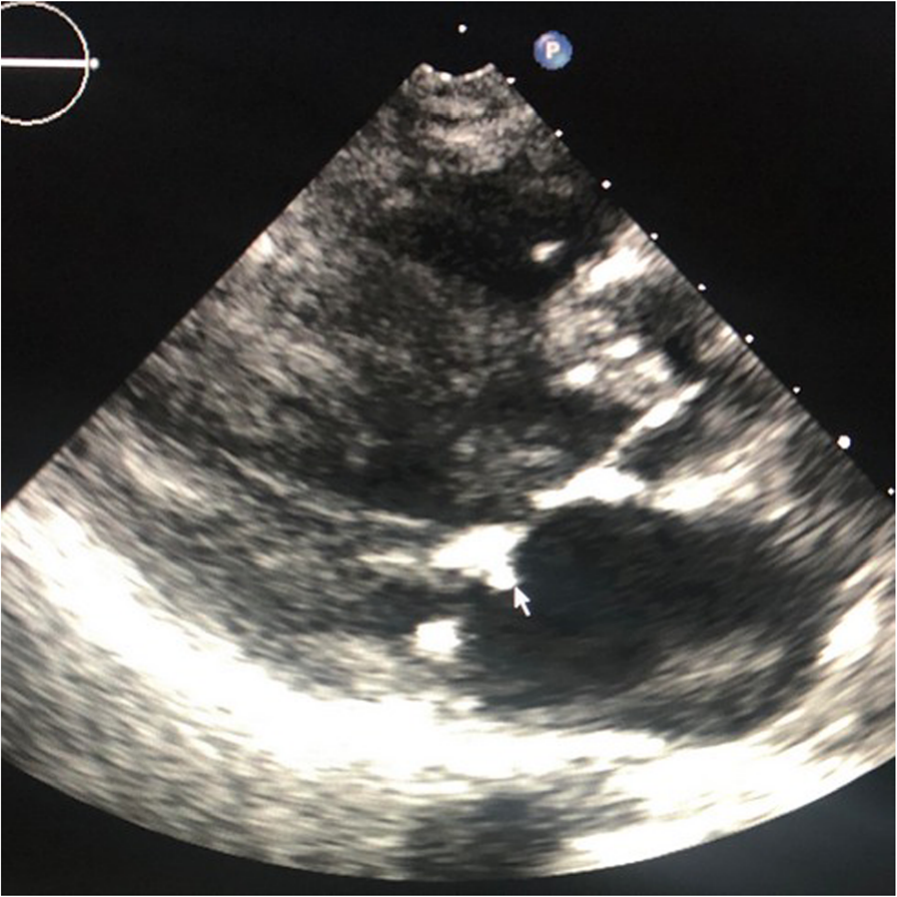
2-D Transthoracic echocardiogram. Parasternal long axis showing vegetation on mitral valve. Indicated by arrow

**Fig. 4:**
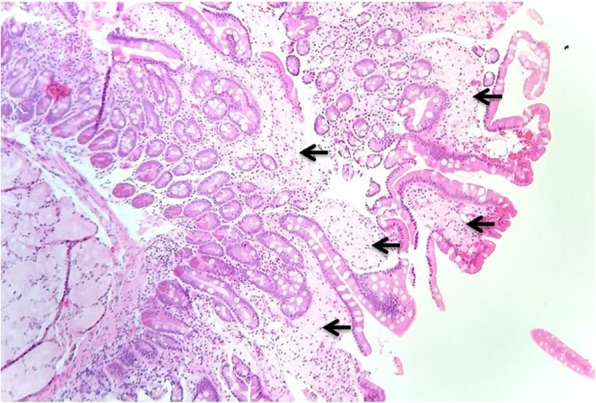
A small bowel biopsy specimen shows expansion of the lamina propria by abundant, pink, foamy macrophages (arrows) (hematoxylin & eosin stain; original magnification × 100)

**Fig. 5:**
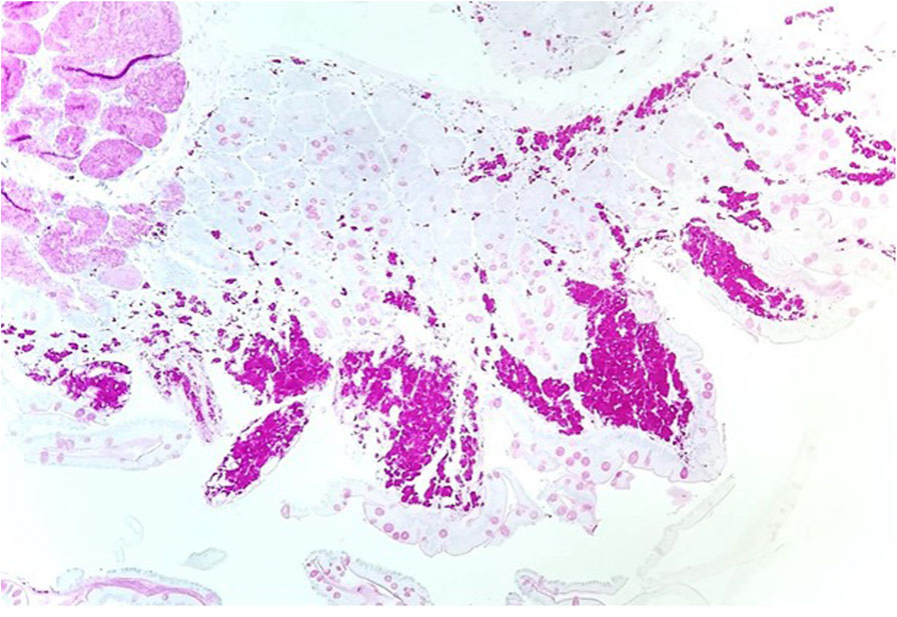
The macrophages in the lamina propria contain PAS positive diastase resistant granules (PAS with diastase stain; original magnification × 100) at arrows

A 56-year-old Caucasian male was first seen as an outpatient with unexplained neutrophilia but otherwise normal complete blood count. He described several years of severe, progressive, refractory, sero-negative migratory arthritis of the large joints along with occasional episodes of indigestion, abdominal pain and diarrhea. At the time of his first visit his laboratory values were significant for elevated erythrocyte sedimentation rate (ESR) at 52 mm/hr., C-reactive protein (CRP) at 7.1 mg/dL, and neutrophilia with white blood cell (WBC) counts at 26.0 × 10^3^/μL. Peripheral blood smear and flow cytometry were done showing no significant abnormalities without atypical cells or blasts. He had been taking celecoxib, methylprednisolone, hydroxychloroquine, and cyclobenzaprine for his arthritis and the neutrophilia was thought to be related to his steroid regimen for arthritis and tobacco smoking. He could not remember how long he had been taking steroids, but stated it was over ‘several years’. He denied alcohol use but admitted to smoking 6 cigarettes per day. He was employed previously as a truck driver. He had a history of depression and anxiety but currently was not in counseling or treated other than cyclobenzaprine. Follow up bone marrow biopsy 2 months later was performed and flow cytometry showed a small population of kappa light chained restricted B lymphocytes but an overall normal cellular marrow with no atypical cells or blasts. At that time his WBC count was 16.0 × 10^3^/μL. He returned 1 month later with new complaints of unintentional 7.2 kg (kg) weight loss with dysphagia. His diarrhea and abdominal pain prompted an esophagogastroduodenoscopy (EGD) and colonoscopy. EGD showed a hiatal hernia, Schatzki ring (which was dilated to 18 mm) and normal appearing duodenum. The gastric mucosa was described to have no active or chronic gastritis seen. Colonoscopy showed hemorrhoids and sigmoid diverticulosis. The small intestine had no diagnostic pathological changes, with no active or chronic enteritis. Biopsies and blood work at that time were unremarkable. Immunohistochemical stains for helicobacter pylori were negative. Histology with periodic acid-Schiff [PAS] staining was not obtained. 7 months later he was readmitted for failure to thrive and a 20.8 kg weight loss. (Fig. 1) The physical examination was remarkable for a cachectic appearing male with II/VI holo-systolic murmur, ecchymosis on upper extremities and scattered petechiae. The remainder of the exam was unremarkable and no lymphadenopathy was detected. Serologic workup showed marked thrombocytopenia with a platelet count at 24,000 and macrocytic anemia, Hg at 7.3 g/dL with MCV (mean corpuscular volume) at 107 fL. Suspicion was high for malignancy yet a repeat bone marrow biopsy was, again, unremarkable with the exception of megakaryocytes suggesting peripheral platelet sequestration as a possible cause for the thrombocytopenia. In addition, ANA (anti-nuclear antibody), Hepatitis and HIV (human immunodeficiency virus) antibodies were negative. Carotid Doppler examination revealed bilateral stenosis and a transthoracic 2D, color echocardiogram revealed no apparent vegetations. He received transfusions of platelets and packed red blood cells (pRBCs) and after a 9 day inpatient admission was discharged with a platelet count of 27,000 and Hg of 7.5 g/dL. He was readmitted 1 month later with a change in mental status according to his roommate but in the same physical state of failure to thrive with continued neutrophilia and worsening thrombocytopenia and anemia. His platelet count was critical at 4000 and Hb was 7.8 g/dL. His WBC was 14.6 × 10^3^/μL. He again received multiunit transfusions of platelets and pRBCs and was started on a higher dose of corticosteroids and intravenous immunoglobulin (IVIG) for presumed underlying autoimmune disease. Psychiatry was also consulted for repeated changes in mood throughout his hospital stay and concluded he had an anxiety disorder. One evening the patient was reported to have garbled speech and decreased right hand grip. A CT (computed tomography) of the head showed subtle hyperdensity in posterior falx and medial left parietal lobe suspicious for subarachnoid hemorrhage. Follow up magnetic resonance imaging (MRI) brain showed multiple extensive bilateral ischemic infarctions (Fig. 2) with areas of post ischemic infarction concerning for infarctions happening at different times. MRA (magnetic resonance angiogram) of the head and neck showed high-grade stenosis of the proximal portion of the right middle cerebral artery and total occlusion of the left internal carotid artery at its origin. These findings suggested a cardiac embolic source. Given his history of esophageal strictures a transthoracic 2D Echo was selected and demonstrated mobile echogenic vegetations (Fig. 3) on the right coronary cusp of the aortic valve, on the aortic surface and on the atrial surface of the anterior mitral leaflet (Fig. 3) near the tip. Serologies were sent for workup of culture negative endocarditis given negative blood cultures to date. After extensive workup the patient was found to have all workup negative with exception of positive *Tropheryma whipplei* PCR (polymerase chain reaction) in the blood. A repeat EGD was performed and small bowel biopsy showed mild villous blunting, distension of lamina propria by an infiltrate of foamy histiocytes (Fig. 4) and PAS positive disease resistant stain (Fig. 5) highlighting well shaped bacteria inclusions confirming diagnosis of WD. The macrophages in the lamina propria were negative for mycobacteria by acid fast stain. The patient was started on ceftriaxone 2GM (grams) daily for 4 weeks with trimethoprim-sulfa DS (double strength) twice daily for at least 1 year. He initially improved but steadily declined and was placed in hospice. 2 months later the hospice nurse noted mental status changes in the few days prior but assured patient remained compliant on medication. The decision was made for the patient to stay in hospice where he expired within the next week. The last platelet count 93,000 the week before his death.

## Discussion and Conclusions

**Fig. 6:**
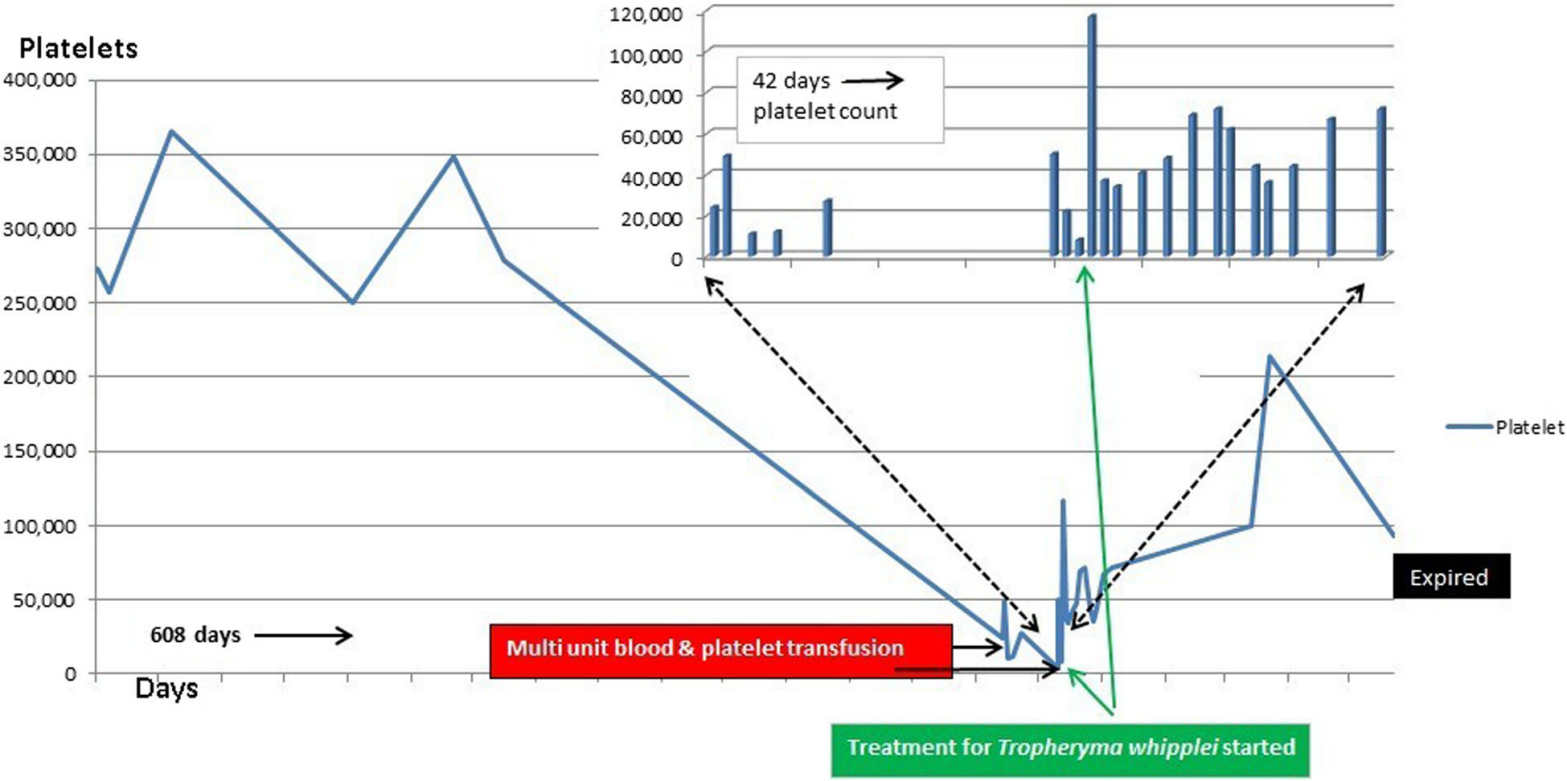
Line graph of platelet count over 608 days. The call-out bar graph shows platelet count during the critical 42 days when the count was lowest and the diagnosis was made. Notice his platelets responded to treatment of the infection

This case is important for four reasons. First, WD is extremely rare with protean manifestations and thus difficult to diagnose often resulting in delays. The late stages of this illness are evident in his last year of life. His prodromal joint pain preceded his generalized symptoms by years and ironically the steroid treatment probably hastened the invasive stage [4]. *T. whipplei* is ubiquitous in the environment and up to 30% of people are colonized [5]. Only a small fraction become infected, however, and it appears that if host immune deficiency is present then progression to invasive disease is more likely to occur. Long term corticosteroid treatment is designed to suppress host immunity, and could also mask symptoms. Patients who are undiagnosed and therefore untreated have an 80% 5-year survival rate after onset of arthralgia, but only 20% 5-year survival after the onset of diarrhea or abdominal pain [3]. It appears to be even lower after the development of thrombocytopenia and endocarditis [3]. 1 month after his initial visit an EGD and colonoscopy were negative but histology with periodic acid-Schiff [PAS] staining was omitted and therefore an opportunity missed. At this point the disease process accelerated to generalized wasting and to include the hematopoietic, cardiovascular and neuropsychiatric organ systems. The second important point of this case is the effect on his platelet count. Approximately 1 year after his initial assessment he was admitted with a platelet count of 4000. In the two previous publications [2, 3] describing thrombocytopenia and endocarditis in WD there was an improvement in the platelet count after treatment started. Our patient likewise had improvement in his platelet count. This suggests that the thrombocytopenia is a direct result of the infection and probably reflects peripheral sequestration that ceases on treatment [3]. In all likelihood profound thrombocytopenia is an end stage finding of this multisystem condition and could be considered a poor prognostic sign when associated with concurrent endocarditis. This patient had the lowest and most persistent thrombocytopenia reported to date as a result of WD. (Fig. 6) The third important component to this case is the development of blood culture negative endocarditis (BCNE), valvular vegetations and embolic stroke with neuropsychiatric dysfunction. He was readmitted shortly after discharge from thrombocytopenia with mental status changes and brain imaging revealed likely embolic events. This led to his echocardiogram which revealed his valvular vegetations. Whether his mental changes were secondary to the embolic events or WD or a combination is unknown but considering the presentation it is more likely the embolic events are directly responsible. CNS (central nervous system) findings have been reported in 10 to 40% of patients with classic WD, however, and include altered mental status and confusion [6]. Nonetheless, the WD was indirectly responsible since the vegetations on the heart valves are the presumed source of the emboli and they were probably caused by *T. whipplei.* Whipple’s endocarditis can be a late constellation of the classic disease or, with greater rarity, present in an isolated form. It usually presents as heart failure or acute ischemic embolic stroke [7], as it did in our patient. Though *T. whipplei* has been noted to involve all layers of cardiac muscle, endocarditis is a diagnostic challenge. Duke’s criteria, though effective in aiding in the diagnosis of infective endocarditis, has severe limitations in the diagnosis of blood culture negative endocarditis (BCNE) and it has been proposed to add PCR analysis of difficult to culture bacteria as a major criterion [8]. BCNE can make up as much as 30% of all infectious endocarditis cases [8]. In fact, in one report, *Tropheryma Whipplei* was found to be the most common cause of BCNE [9]. PCR has been validated in multiple studies to assist in the diagnosis of WD and Whipple endocarditis [9]. PCR testing is a useful adjunct diagnostic tool when Whipple disease cannot be confirmed with PAS positive stained tissue, however in tissues with PAS positivity, PCR and immunohistochemistry, though helpful, are only necessary in select cases [3] Indeed, our patient first had a positive noninvasive blood PCR followed by a targeted small bowel biopsy and stain. The fourth important component of this case concerns the treatment. Treatment of WD remains controversial. Prior to the ability to culture *Tropheryma Whipplei*, successful treatment was also determined using clinical judgment and disappearance of previously positive tests such as PCR. Many cases were treated with induction therapy of different antibiotics, such as benzylpenicillin 1.2 million units and streptomycin 1GM daily for 2 weeks, with a seemingly agreed upon maintenance therapy of trimethoprim-sulfamethoxazole for at least 1 year [10]. However, recent publications have noted several cases of relapse on this regimen and note the preferred therapy to be a combination of doxycycline and hydroxychloroquine [11, 12]. One study was able to test for antibiotic susceptibility and show ceftriaxone and trimethoprim-sulfamethoxazole to be ineffective entirely [12]. Testing the cerebrospinal fluid for the PCR seems to be agreed upon as symptoms are often masked and this can help ensure treatment, eradication and also detect relapse [13]. Unfortunately, our patient had a late presentation of CNS symptoms, while in hospice and he died shortly after. Thus, without CSF, CNS biopsy or post-mortem examination we can only suspect he may have had CNS involvement of T. Whipplei. However, this also brings an important point to the possibility of IRIS (Immune Reconstitution Inflammatory Syndrome). IRIS has been well described in patients on long term immunosuppressive therapy and, though an autopsy was not done on our patient, this could be a potential explanation behind his demise despite appropriate treatment and clinical response [14]. IRIS occurs secondary to an abnormal reconstitution of the immune system once immune suppression has resolved and is well described in numerous diseases causing immune suppression or in therapy inducing immune suppression. IRIS is strongly associated with long term immunosuppressive therapy prior to the diagnosis of Whipples disease and can be seen in patients initially misdiagnosed to have rheumatoid arthritis and subsequently started on antibiotics and discontinued on immunosuppressive therapy [14]. Strangely enough, in the event this occurs, starting patients back on steroid treatment is the treatment when inadequate response to appropriate treatment is suspected [14]. Whipple’s endocarditis, though oftentimes treated with surgery, has been treated successfully without surgical resection of valve tissue [15].

This case is notable for several reasons. First, WD is exceedingly rare and frequently thought of too late. Due to this, our patient went undiagnosed for over 1 year, and ultimately developed critical thrombocytopenia. The association between WD, endocarditis and thrombocytopenia is probably a function of the duration of time the disease has been actively invasive [2, 3]. Second, this case revealed a challenge in the ability for Duke’s criteria to diagnose BCNE [8]. Our case had an echocardiogram positive for IE and arterial emboli which, according to the 2000 criteria would be a “possible case”. Nonetheless, there is clearly a need to introduce more comprehensive criteria for fastidious bacteria, and these should include now widely available molecular methods. Finally, there is no agreed upon treatment regimen and even those that have been reported in numerous reports are slowly becoming obsolete in the literature. Our patient expired under the previously widely accepted treatment of ceftriaxone induction therapy and trimethoprim/sulfamethoxazole maintenance therapy. This could possibly be attributable to a growing resistance or the collective damage already delivered by the disease.

## Data Availability

Data sharing is not applicable to this article as no datasets were generated or analyzed during the current study.

## Abbreviations

ANA: Anti-nuclear antibody
BCNE: Blood culture negative endocarditis
CNS: Central nervous system
CRP: C-reactive protein
CT: Computed tomography
DS: Double strength
EGD: Esophagogastroduodenoscopy
ESR: Erythrocyte sedimentation rate
GM: Grams
HIV: Human immunodeficiency virus
IVIG: Intravenous immunoglobulin
MCV: Mean corpuscular volume
MRA: Magnetic resonance angiogram
MRI: Magnetic resonance imaging
PAS: Periodic acid-Schiff
PCR: Polymerase chain reaction
pRBCs: Packed red blood cells
WBC: White blood cell
WD: Whipple’s disease
